# An Efficient In Vitro Regeneration Protocol and the Feature of Root Induction with Phloroglucinol in *Paeonia ostii*

**DOI:** 10.3390/plants13223200

**Published:** 2024-11-14

**Authors:** Keyuan Zheng, Luming Yao, Yumei Xie, Shuiyan Yu, Yonghong Hu, Mulan Zhu

**Affiliations:** 1 Shanghai Key Laboratory of Plant Functional Genomics and Resources, Shanghai Chenshan Botanical Garden, Shanghai 201602, China; kyzheng@cemps.ac.cn (K.Z.);; 2 National Key Laboratory of Plant Molecular Genetics (NKLPMG), CAS Center for Excellence in Molecular Plant Sciences, Shanghai 200032, China; 3 Department of Plant Science, School of Agriculture and Biology, Shanghai Jiao Tong University, No 800, Dongchuan Rd., Shanghai 200240, China; lmyao@sjtu.edu.cn

**Keywords:** *Paeonia ostii*, adventitious shoots regeneration, in vitro root induction, phloroglucinol, molecular signature

## Abstract

*Paeonia ostii*, a plant of substantial economic significance, continues to face constraints in achieving large-scale propagation. In vitro propagation offers a promising avenue for the production of disease-free plants and the genetic transformation of peonies to instill novel traits. However, significant challenges persist in tissue culture, particularly with regards to the reproduction coefficient of shoots and the rooting process. This study reports an efficacious protocol for *P. ostii* micropropagation, focusing on in vitro root development facilitated through the application of phloroglucinol (PG). Furthermore, the study unveils the molecular signature of *P. ostii* during in vitro root development. The results indicate that the modified Y3 medium (Y3M), supplemented with 1 mg/L 6-benzyladenine (BA) and 0.1 mg/L α-naphthaleneacetic acid (NAA), is optimal for adventitious bud induction, achieving a 96.67% induction rate and an average of 16.03 adventitious shoots per sample. The highest elongation percentage (92.15%) and the longest average shoot length (3.87 cm) were obtained with Y3M containing 0.3 mg/L BA and 0.03 mg/L NAA. Additionally, the optimal medium for inducing root formation in *P. ostii* was identified as WPM supplemented with 3 mg/L indole-3-butyric acid (IBA) and 100 mg/L phloroglucinol (PG). Lignin content detection, microscope inspection, and molecular signature results demonstrated that PG enhanced lignin biosynthesis, thereby promoting in vitro rooting of *P. ostii*.

## 1. Introduction

*Paeonia ostii* ‘Feng Dan’ is a widely cultivated tree peony (*Paeonia suffruticosa* Andr.) in China, esteemed for its medicinal, ornamental, and oil-producing properties [1]. The root bark, known as Mu Dan Pi, is utilized in traditional Chinese medicine to treat various ailments, including cardiovascular inflammation, allergies, and neurodegenerative disorders [2,3]. Notably, the seeds of ‘Feng Dan’ are rich in unsaturated fatty acids, making them a valuable source for edible oil production [4,5]. The flowers exhibit antioxidant properties and were officially recognized as a food material by the Chinese Ministry of Health in 2013 [2,6,7]. Additionally, natural active compounds such as paeoniflorin and paeonol, isolated from peony extracts, exhibit significant medicinal value and hold promise for the development of novel pharmaceuticals [8,9,10]. Given these diverse and valuable attributes, establishing sustainable propagation systems for *Paeonia ostii* is imperative.

The limitations inherent in traditional propagation techniques, including seeding, division, and grafting, have significantly restricted the advancement of tree peony breeding and propagation [11,12,13]. The dormancy period required for sowing and propagation spans up to 2 years in tree peonies. Coupled with the highly heterozygous genetic backgrounds of tree peony [14], this results in substantial variations among propagated seedlings, challenging the preservation of desirable hereditary traits. Propagation by division has a long reproduction cycle, low reproduction coefficient, and a tendency to accumulate viruses, leading to a decline in germplasm quality. Grafting, on the other hand, is a complex, time-consuming procedure that is vulnerable to environmental conditions. Therefore, there is an imminent requirement for alternative and efficient large-scale propagation methods in tree peony cultivation.

The in vitro propagation process allows for the rapid production of a large number of genetically identical plants in a short period, facilitating the mass production of disease-free tree peonies and enabling genetic transformation for novel trait development. Among in vitro methods, direct organogenesis was selected for this study to address specific challenges encountered in *P. ostii* tissue culture. Direct organogenesis minimizes the risk of genetic variation associated with callus formation in indirect organogenesis, making it particularly suited for the propagation of genetically uniform seedlings. Considering the need to preserve the specific genetic traits of *P. ostii*, direct organogenesis provides an efficient approach to achieve rapid shoot proliferation while enhancing genetic stability.

To date, only a handful of cultivars have been established comprehensive regeneration systems for tree peony, such as *P. ostii* ‘Feng Dan’, *P. sufruticosa* ‘Jin Pao Hong’, ‘Wu Long Peng Sheng’, *P. × lemoinei* ‘High Noon’, etc. [15,16,17,18,19,20,21]. Micropropagation of *P. ostii* primarily involves callus induction and bud proliferation, with multiplication coefficients and rooting remaining as the bottleneck of tissue culture [17,20,22,23]. Although several rooting media and protocols have been reported for *P. ostii*, inducing adventitious roots in numerous cultivars remains challenging. Even upon successful induction of adventitious roots, issues such as low rooting rates, underdeveloped root systems, and difficulties in acclimatization persist.

Phloroglucinol (PG), a phenolic compound generated through the degradation of phloridzin, is commonly employed as a supplementary component alongside other plant growth regulators in vitro. PG functions as an auxin synergist or protector, effectively augmenting both shoot multiplication and root development [24,25,26,27,28]. As a media additive, it significantly enhances shoot bud formation in nodal cultures and promotes the in vitro development of tuberous roots in micro shoots, as evidenced in *Tinospora cordifolia* [29]. In an investigation conducted by Londe et al., a PG concentration of 200 µM was examined for its capacity to promote in vitro root multiplication and elongation in *Musa accuminata* cv. Grand Naine [30], further highlighting the versatile application of PG in plant tissue culture. Moreover, PG prevents hyperhydricity by enhancing the activity of enzymes in the lignin biosynthesis pathway [27]. However, the molecular mechanisms underlying PG’s role in plant tissue culture remain elusive, and its application specifically in tree peony tissue culture has yet to be explored.

Here, we present an effective protocol for tree peony micropropagation, particularly focusing on rooting in vitro with PG application, and elucidate the molecular signature underlying peony root development in vitro.

## 2. Results

### 2.1. Effect of PGRs on Shoot Regeneration

**Figure 1:**
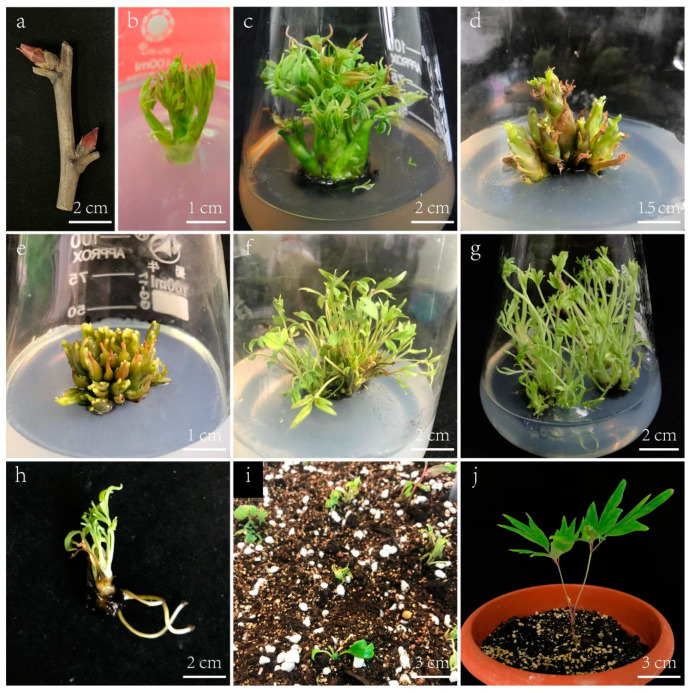
In vitro regeneration system of *P. ostii*. (**a**) Axillary shoots of *Paeonia ostii* ‘Fengdan’. (**b**) Axillary shoots initially cultured for 5 days. (**c**) Axillary shoots initially cultured for 20 days. (**d**) Regeneration adventitious shoots (cultured for 30 days). (**e**) Regeneration adventitious shoots (cultured for 60 days). (**f**) Elongation of adventitious shoots cultured for 20 days. (**g**) Elongation of adventitious shoots cultured for 35 days. (**h**) Roots of regenerated plantlets. (**i**) Transplanted regenerated plants. (**j**) The plantlets after acclimatization for 1 year.

After disinfection, axillary shoots of *P. ostii* (Figure 1a) were prepared by removing the bud scales and initiating culture (Figure 1b). When new axillary shoots reached 1–2 cm in length (Figure 1c), they were excised and transferred to an adventitious shoot regeneration medium.

To determine the optimal combination of PGRs for adventitious shoot regeneration, axillary shoots measuring 1–2 cm in length were cultured under nine distinct treatment conditions. After 15 days of cultivation in the medium, small adventitious shoots began to emerge around the axillary shoots. Over the course of 30 days, the gradual induction of adventitious shoots became evident (Figure 1d). Subsequently, the shoots were subdivided and returned to the same medium as the first stage for an additional 30 days to enhance the induction of adventitious shoots.

A comprehensive analysis of the outcomes, presented in Table 1, highlighted the significance of PGR concentrations. Extreme concentrations of BA and NAA, either excessive or deficient, were unfavorable for inducing adventitious shoots. These extremes resulted in low induction rates and a limited number of differentiated adventitious shoots. The most promising results were achieved with a concentration of 1 mg/L BA and 0.1 mg/L NAA, as illustrated in Figure 1e. This particular combination yielded the highest adventitious shoot induction rate at 96.67% and an average of 16.03 adventitious shoots per sample.

### 2.2. Effects of PGRs on Shoot Elongation

To identify the optimal combinations and concentrations of PGRs for promoting shoot elongation, adventitious shoots were cultured on Y3M medium fortified with various combinations of BA and NAA (Table 2). After a 20-day period, most adventitious shoots exhibited elongation (Figure 1f). To further promote elongation of adventitious shoots, the shoots were divided into smaller clusters and re-cultured on the initial medium for an additional 15 days. Among the tested combinations, the highest elongation percentage (92.15%) and longest average shoot length (3.87 cm) were achieved on Y3M containing 0.3 mg/L BA and 0.03 mg/L NAA (Figure 1g). Closely following this was the medium supplemented with 0.3 mg/L BA and 0.01 mg/L NAA, which exhibited an elongation percentage of 90.6% and an average shoot length of 3.11 cm.

### 2.3. Effect of PG on Root Induction

**Figure 2:**
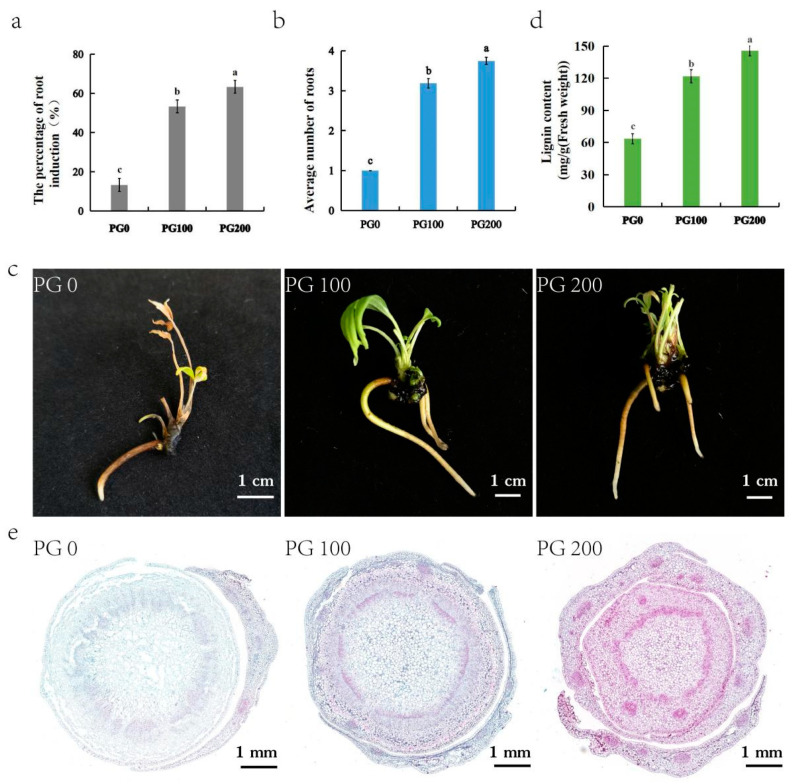
Effect of PG application on *P. ostii* rooting. (**a**) The percentage of rooting. (**b**) Average number of roots. (**c**) Seedling rooting status with PG application. (**d**) The results of lignin content detection. (**e**) Microscopic observation of stained sections. Different lowercase letters within the same image indicate significant differences.

In this study, a series of experiments were conducted to investigate the induction of adventitious roots during the in vitro regeneration of *P. ostii*. The findings revealed varying degrees of root induction among three treatments, primarily influenced by the presence and concentration of PG in the culture medium. The results demonstrated significant enhancement in root induction with the incorporation of PG into the medium, as depicted in Figure 2a–c. Specifically, the addition of PG at concentrations of 100 mg/L and 200 mg/L significantly facilitated peony root formation. In contrast, without PG supplementation, the rooting rate was only 13.33%, with limited root development, feeble growth, and poor post-transplant survival rates.

The highest root induction percentage, at 63.33%, and an average of 3.75 roots per explant were observed in the medium containing 200 mg/L PG. However, the aboveground growth was suboptimal, with stunted leaf development (Figure 2c), and the survival rate after transplantation was low, approximately 50%. Conversely, the medium enriched with 100 mg/L PG demonstrated a root induction rate of 53.33% and an average of 3.19 roots per explant. Plants cultured in this medium exhibited robust growth and strong vitality. After acclimatization and transplantation, the survival rate of rooted seedlings was significantly higher, at around 85% (Figure 1i,j).

In conclusion, these findings suggest that the addition of 100 mg/L PG to the culture medium represents the optimal concentration for inducing root formation in *P. ostii*.

### 2.4. Lignin Profile During In Vitro Root Induction with PG

The lignin profiles of the three root induction treatment groups showed different patterns (Figure 2d). A lignin content of 145.96 mg/g was observed in the medium supplemented with 200 mg/L PG, followed by 121.99 mg/g in the medium containing 100 mg/L PG. Both treatments showed higher lignin content compared to the medium lacking PG.

To further investigate lignification, saffron and fast green staining were applied to the stems from the three rooting treatments. The lignified cell walls stained red, while the non-lignified regions remained green. The staining patterns revealed significant differences among the treatments (Figure 2e). The number of lignified cells of the *P. ostii* stem cultured on the medium with PG was outstandingly higher than those grown in the absence of PG. The number of red cells in stems cultured with 200 mg/L PG was slightly elevated compared to those cultured with 100 mg/L PG.

### 2.5. Global Analysis of Gene Expression with PG Application

**Figure 3:**
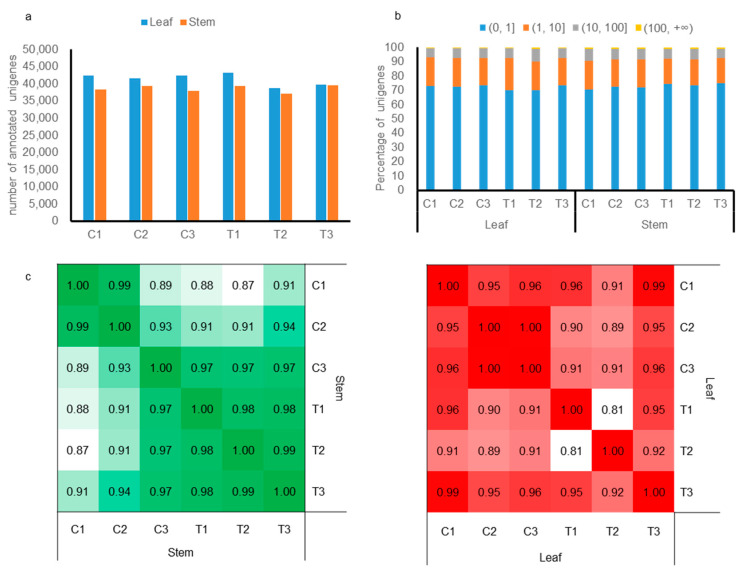
Overview of unigene expression in leaves and stem of *P. ostii* with PG application. (**a**) Total number of annotated unigenes in each library. (**b**) Proportion of unigenes with indicated expression strength at each level (TPM). (**c**) Pearson correlation coefficients (PCCs) of gene expression (TPM) between different treatments. The PCCs of expressed unigenes from leaves and stems were calculated separately.

To investigate global gene expression dynamics in tree peonies following in vitro PG treatment, stem and leaf tissue samples were harvested from plants cultured in WPM medium supplemented with 100 mg/L PG for 30 days. Samples were collected in triplicate for analysis. Subsequently, twelve libraries were prepared, yielding an average of 113.5 million raw reads each, of which approximately 84.7 million reads per library were successfully mapped (Table S1). A total of 104,838 unigenes were assembled across all libraries, averaging 1160.02 bp in length, and 49,092 unigenes were annotated with comparable sequences in all libraries (Figure 3a, Table S2).

The expression levels of unigenes, normalized to transcripts per million (TPM) reads, demonstrated broad uniformity across all treatment groups, as depicted in Figure 3b and detailed in Table S3. Notably, within either leaves or stems, expression profiles between any two libraries displayed substantial correlation (average correlation coefficient: r > 0.81; for leaves: 0.87, and for stems: 0.81). This observation implies that a relatively constrained subset of unigenes was implicated in the response to PG treatment, whereas the expression patterns of the majority of unigenes remained consistent, as illustrated in Figure 3c.

### 2.6. Differential Regulation of Unigenes of P. ostii During In Vitro Root Induction with PG

**Figure 4:**
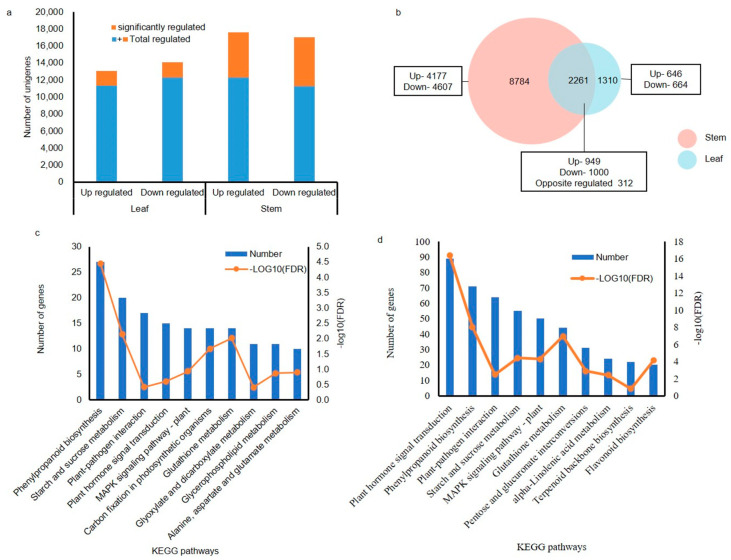
Differential regulation of unigenes in *P. ostii* with PG application. (**a**) Number of differential regulation unigenes. Total regulated means: |log2FC| > 0, significantly regulated means: |log2FC| ≥ 1. (**b**) Venn diagram of DEGs (|log2FC| ≥ 1) in leaf and stem with phloroglucinol treatment. Opposite regulated means the regulation trends were opposite in the leaf and stem. (**c**) Top 10 significantly enriched KEGG pathways in leaves, with adjusted *p*-values (Padjust or false discovery rate, FDR) < 0.05. (**d**) Top 10 significantly enriched KEGG pathways in stems, with adjusted *p*-values (Padjust or false discovery rate, FDR) < 0.05.

To elucidate the unigenes differentially regulated by PG, their expression patterns were analyzed via DESeq. This analysis revealed a total of 3571 and 11,045 significantly regulated unigenes (|log2FC| ≥ 1) in the leaves and stems, respectively. Remarkably, the distribution of up- and down-regulated unigenes was balanced in both samples (Figure 4a). Moreover, a common subset of 2261 differentially expressed genes (DEGs) was identified in both stems and leaves (Figure 4b). These DEGs underwent further characterization through annotations using the Gene Ontology (GO) and Kyoto Encyclopedia of Genes and Genomes (KEGG) databases (Datasets S1 and S2).

To explore the potential function of DEGs in both leaves and stems under PG treatment, GO and KEGG enrichment analyses were conducted. Overall, the enriched GO items were similar between leaves and stems, indicating a comparable response to PG. However, within the top 20 significantly enriched GO items, only stems exhibited enrichment for biological processes related to auxin (response to auxin and auxin-activated signaling pathway) and lignin (Figures S1 and S2). In KEGG enrichment analysis, pathways involved in phenylpropanoid synthesis and plant hormone signal transduction were significantly enriched in both tissues. However, the number of enriched pathways was notably higher in stems compared to leaves (Figure 4c,d).

### 2.7. Expression Patterns of Genes Related to Phytohormone Signal Transduction

The plant hormone signal transduction pathways were significantly enriched during PG treatment. Among the DEGs, a total of 96 related genes were identified, including 47 auxin-related genes, 13 cytokinin-related genes, and 9 brassinosteroid-related genes, all playing crucial roles in cell division and enlargement (Dataset S3).

**Figure 5:**
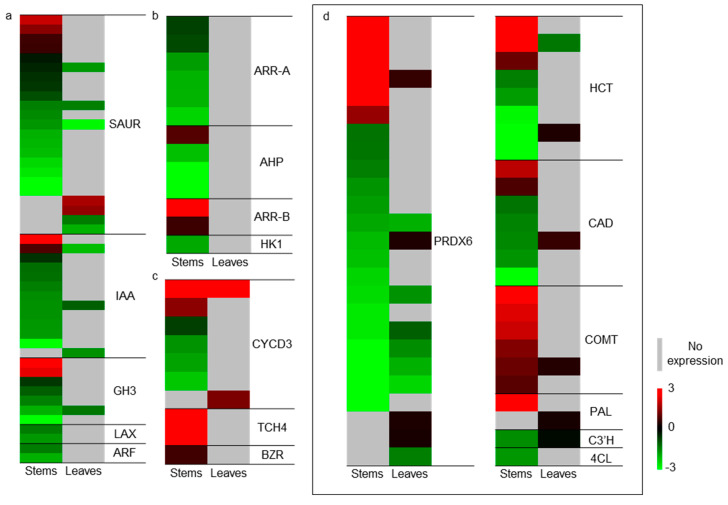
Heat map visualizes the expression patterns of genes related to phytohormone signal transduction (**a**–**c**) and lignin biosynthesis (**d**). The expression data were normalized with transcripts per million reads (TPM) from RNA-seq data.

Hierarchical clustering of auxin-related genes—specifically those encoding Small Auxin Up-Regulated RNA (SAUR) proteins, Indole-3-Acetic Acid-Inducible (AUX/IAA) proteins, Auxin-Responsive GH3 Family Proteins (GH3), Like-Auxin Resistant (LAX), and Auxin Response Factors (ARFs)—demonstrated a predominant down-regulation of these genes (Figure 5a).

Within the differentially expressed genes (DEGs), 13 cytokinin-related genes were identified: six Response Regulator (ARR-A) genes, four Histidine-Containing Phosphotransmitter (AHP) genes, two Pseudo-Response Regulator (ARR-B) genes, and one Histidine Kinase 1 (HK1) gene. Most of those genes were down-regulated, except ARR-B, which serves as a negative regulator of cytokinin signaling (Figure 5b). With regard to the brassinosteroid related genes, the genes involved in Brassinazole-Resistant (BZR) and endoxyloglucan transferase 4 (TCH4) were up-regulated, while most genes encoding cyclin-dependent protein kinase 3 (CYCD3) were repressed (Figure 5c).

### 2.8. The Expression Changes of Genes Involved in Phenylpropanoid Biosynthesis

The KEGG analysis highlighted a significant enrichment in phenylpropanoid biosynthesis pathways, implicating 71 DEGs in these processes. Of these, 50 genes were specifically associated with lignin biosynthesis, including those encoding Peroxidase 6 (PRDX6), Hydroxycinnamoyl-COA Shikimate/Quinate Hydroxycinnamoyl Transferase (HCT), Cinnamyl-Alcohol Dehydrogenase (CAD), Caffeic Acid 3-O-Methyltransferase (COMT), Phenylalanine Ammonia-Lyase (PAL), 4-Coumarate-Coa Ligase (4CL) and 5-O-(4-Coumaroyl)-D-Quinate 3′-Monooxygenase (C3′H) (Dataset S4). The majority of the genes were down-regulated, except COMT and PAL, which were both up-regulated (Figure 5d).

Additionally, the potential associations between proteins involved in phytohormone (auxin, brassinosteroids (BR), and cytokinin (CK)) signal transduction and lignin biosynthesis pathways were analyzed. Despite observed interactions among proteins associated with plant hormone synthesis, analysis revealed that phytohormone signal transduction pathways and lignin biosynthesis operate independently in the presence of PG treatment (Figure S3).

## 3. Discussion

*P. ostii* is characterized by extensive cross-pollination and seed propagation, resulting in a highly heterozygous cultivated variety group [14]. Consequently, individual seedlings exhibit significant trait variations, including differences in fertility [31]. To ensure the stable genetic inheritance of superior genotypes, we established an efficient in vitro propagation system using *P. ostii* ‘Feng Dan’ axillary shoots as explants. However, challenges such as low propagation coefficients and poor rooting frequently arise during in vitro propagation [32,33].

The Y3M medium helps to mitigate hyperhyidricity and enhances the quality of regenerated shoots. Traditionally, MS and WPM media are commonly used for *Paeonia* regeneration [15]. In our study, axillary shoots were initially cultured on WPM medium supplemented with 2.0 mg/L BA and 0.2 mg/L NAA. However, signs of hyperhydricity were observed. To address this, the Y3M medium was used in subsequent adventitious shoot induction experiments. Previous studies have reported that increasing the concentration of Ca(NO_3_)_2_ in WPM medium, which elevates calcium (Ca^2+^) levels, promotes the proliferation and growth of in vitro plantlets [23]. The distinctive feature of the Y3M medium is its high potassium ion content (Table 3). Potassium (K+) is more stable than Ca^2+^ in cell sap and is the most suitable cation for controlling vital functions [34]. Research indicates that potassium application can enhance photosynthetic rates, as it is essential for various physiological processes that directly impact photosynthesis [35]. Moreover, potassium is critical for plant water relations, influencing photosynthesis, transpiration rates, and stomatal control [36]. By combining the Y3M medium with PGRs, our study significantly increased the number and quality of regenerated shoots, achieving a propagation coefficient of up to 16. These high-quality regenerated shoots appear to support subsequent in vitro root induction.

PGRs are essential in establishing an effective regeneration system for peonies. In existing studies, the cytokinin BA is frequently used as the primary hormone for adventitious shoot induction, often in combination with hormones such as NAA, GA_3_, and 2,4-D [15,16,19]. The proliferation coefficient varies by cultivar and specific culture conditions, generally ranging from 3.0 to 5.0. In previous regeneration studies of *P. ostii* ‘Feng Dan’ by Wang et al. and Liu et al., BA and GA_3_ were used as the primary hormones for adventitious shoot proliferation, achieving proliferation coefficients of 3.9 and 5.4, respectively [17,23]. In this study, BA and NAA were used, resulting in an optimal proliferation coefficient of 16, substantially exceeding previously reported values for *P. ostii* ‘Feng Dan’. Additionally, the study shows the importance of hormone ratios in promoting adventitious bud proliferation and elongation, with a 10:1 ratio proving optimal, while a 5:1 ratio also showed a strong induction effect (Table 1), aligning with the author’s previous findings on mango regeneration [37].

PG is effective in plant tissue culture, especially in enhancing the in vitro rooting of ligneous plants [38,39,40], serving as a hormone-like supplement. In this study, PG significantly increased the rooting rate of tree peony seedlings, and the expression of genes was analyzed to gain better understanding of the physiological mechanisms underlying this process. Although the number of annotated unigenes in both leaves and stems was comparable (Figure 3a), many more DEGs were detected in stems (leaves: 3571, stems: 11,045) (Figure 4a). These findings suggest that stems, where root initiation and development occur, might be more sensitive to PG, possibly explaining its impact on rooting in plant tissue culture [24,41]. Additionally, distinct phenotypic changes in rooting were observed with PG application (Figure 2). Nevertheless, the correlation coefficient between PG treatment and control (mock) was remarkably high (Figure 3c), suggesting that a limited subset of genes participates in the response to PG during rooting, whereas the expression patterns of the majority of genes remain constant. A similar high correlation was also reported in *Cariniana legalis* during rooting of in vitro shoots with PG treatment, where only 334 differential proteins were identified [39].

PG can mitigate challenges by providing precursors and increasing the activity of enzymes related to lignin biosynthesis [31,42]. In this study, the content and distribution of lignin in stems significantly increased upon application of PG (Figure 2d,e). Furthermore, most genes involved in lignin biosynthesis pathways were significantly regulated (Figure 5d). Upon application of PG to tree peony, most genes related to lignin biosynthesis showed reduced expression, while others remained unchanged. This indicates a negative relationship between root induction and lignin synthesis. Similar observations were made in Cariniana legalis during rooting with PG treatment, where the enzymes phenylalanine ammonia-lyase, caffeoyl-CoA O-methyltransferase, and chalcone synthase showed decreased accumulation [41]. Among the DEGs, 31 out of 50 genes were related to COMT and PRDX6, which encode the last two key enzymes in the lignin biosynthesis pathway [43]. All genes encoding COMT were up-regulated, suggesting that PG may serve as a precursor or substrate for COMT and PRDX6, enhancing the final steps of lignin synthesis while limiting upstream enzyme activity.

PG acts solely as a hormone-like supplement. Auxin, cytokinin, and brassinosteroid are classes of phytohormones that regulate a variety of developmental processes, including cell division and elongation and shoot and root initiation. Exogenous phenolic compounds, such as PG, function as alternative substrates for oxidative catabolism, protecting phytohormones from oxidative breakdown [42]. In peony treated with PG, the genes involved in phytohormone signal transduction (specifically axiun, cytokinine, and brassinosteroid) were significantly regulated and the expression of most genes was reduced (Figure 5a–c). This suggests that the hormone regulation during root initiation and development was inhibited. This finding may not align with the hypothesis that phenols, such as PG, synergize with phytohormones and protect them from breakdown [27]. Regeneration of *Ornithogalum dubium* was observed in the circumstance of PG alone, demonstrating auxin/cytokines-like properties, and it indicated that PG acted as a hormone-like molecule and triggered direct rooting without callus formation [26]. Similarly, in peony, PG can serve as a sole hormone-like supplement, mimicking the functions of auxin, cytokines, and brassinosteroid while inhibiting endogenous hormone signal transduction.

PG acts as a versatile component in plant growth and development. PG, a precursor in the lignin biosynthesis pathway, effectively mitigates hyperhydricity through lignification and enhances the rooting rates when used in conjunction with phytohormones or as a hormone-like molecule in several horticultural plants [25,26,38,39]. The interaction between lignin biosynthesis and hormone signal transduction pathways was analyzed, but no direct relationship was revealed (Figure S3). This suggests that PG may have diverse roles in plant growth and development, potentially engaging with multiple pathways and mechanisms.

## 4. Materials and Methods

### 4.1. Plant Materials and Treatments

Axillary shoots of tree peony cultivars *P. ostii* ‘Fengdan’ (Figure 1a) were randomly collected at the beginning of sprouting in spring from healthy plants from the Shanghai Chenshan Botanical Garden, Songjiang District, Shanghai, China.

The shoots underwent a thorough cleansing process in running tap water for 1–2 h. Subsequently, sterilization procedures were carried out, involving 30 s of immersion in 75% *v*/*v* ethanol, followed by a 10 min treatment in a solution of 1% *v*/*v* benzalkonium bromide sterilization. The sterilized shoots then underwent three successive rinses with sterile distilled water, concluding with a 10 min immersion in 50% *v*/*v* Plant Preservative Mixture (Yeasen Biotechnology (Shanghai) Co., Ltd., Shanghai, China). After disinfection, the bud scales were excised and used as explants for micropropagation.

### 4.2. Basal Medium and Growth Conditions

In this study, we utilized two different media (Table 3): (i) Woody Plant Medium (WPM) [44] and (ii) Y3M (modified Y3 medium). The Y3M medium is a modification of the standard Y3 medium [45].

The in vitro container used 100 mL glass Erlenmeyer flasks, each filled with about 40 mL of media. The media were adjusted to a pH of 5.8, solidified with 6 g/L agar (ShiZe Biotechnology (Shanghai) Co., Ltd., Shanghai, China), and autoclaved at 121 °C for 18 min. For shoot bud induction and elongation, the formulation included 30 g/L sucrose, whereas the rooting medium contained 20 g/L sucrose. Cultures were kept in the laboratory at 24 ± 2 °C with a 16/8 h light/dark cycle using cool white fluorescent illumination (1500–3000 lx), except during root induction experiments.

### 4.3. Induction of Adventitious Shoots

Following sterilization, the shoots were initially cultured on WPM containing 2.0 mg/L 6-benzyladenine (BA) and 0.2 mg/L α-naphthaleneacetic acid (NAA) (Figure 1b). When the axillary shoots grew to 1–2 cm in length (Figure 1c), they were harvested and moved to Y3M medium with different concentrations of BA (0.5, 1.0, 2.0 mg/L) and NAA (0.05, 0.1, 0.2 mg/L) to stimulate adventitious bud formation. A total of nine treatments were conducted, each with three replications and a total of 60 explants. After a 60-day period, the percentage of bud induction and the adventitious shoot number were recorded.

### 4.4. Elongation of Shoots

To promote shoot elongation in *P. ostii*, a two-stage approach was employed. Initially, adventitious shoots were cultured on Y3M medium with varying concentrations of BA (0.1, 0.3, 0.5 mg/L) and NAA (0.01, 0.03, 0.05 mg/L) for 20 days. The elongated shoots were then divided into small clusters and returned to the same medium for an additional 15 days. Each treatment was replicated three times, yielding a total of 60 explants. After a 35-day incubation period, the percentage of shoot elongation was calculated, and the lengths of the elongated shoots were measured.

### 4.5. Rooting of Adventitious Shoots

The elongated shoots were excised, transferred to an adventitious root induction medium, and divided into three treatment groups. The first group was placed on WPM medium with 3 mg/L indole-3-butyric acid (IBA). The second group was cultured on WPM medium with 3 mg/L IBA and 100 mg/L phloroglucinol (PG), while the third group was placed on WPM medium with 3 mg/L IBA and 200 mg/L PG. Each treatment was replicated three times, yielding a total of 30 explants. After a 60-day incubation period in the dark followed by 10 days in the light, the percentage of root induction, average root length, and average number of roots were meticulously assessed and recorded for each group.

### 4.6. Acclimatisation and Transplantation

The bottles containing well-developed root plantlets were transferred to the greenhouse. A 1 cm layer of sterile water was added to each bottle, and the caps were left open. Following this, the plantlets underwent a 7-day acclimation period in the greenhouse environment before transplantation. Subsequently, the plantlets were transferred to a disinfected substrate consisting of peat soil, perlite, and vermiculite in a 2:1:1 volume ratio, with disinfection achieved using a 0.1% *v*/*v* potassium permanganate solution. Following thorough watering, the plantlets were enveloped in transparent plastic film to maintain humidity and air exchange, which was replenished daily. After a period of 15 days, the film was removed to commence routine watering and fertilization management.

### 4.7. Lignin Content Detection

The shoots were cultured on the adventitious root induction medium supplemented with varying concentrations of PG (0, 100, 200 mg/L) for 30 days. Subsequently, the stems were harvested, and the lignin content was assayed using a dedicated kit. A standard curve was established between lignin concentration and OD450 absorbance according to the kit operating instructions and utilizing standard sample concentration. The sample stem was cut into small pieces, ground, and weighed. Then, 0.9 mL PBS buffer was added to the centrifuge tube, mixed well, and centrifuged to obtain the lignin solution from the supernatant. The remaining steps were carried out in accordance with the ELISA detection kit operation (Beijing Bio-Tech Pack Technology Co., Ltd., Beijing, China). The optical density at 450 nm (OD450) was measured, and the lignin concentration was determined using a standard curve. Then, the amount of lignin per gram of fresh weight in the plant sample was determined.

### 4.8. Safranin Fast Green Staining

The shoots were cultured for 30 days under three different rooting treatments, after which 5 mm stem base segments were collected for paraffin sectioning. The sections were first immersed in xylene (Sinopharm Chemical Reagent Co., Ltd., Shanghai, China) I for 20 min, followed by xylene II for 20 min. They were then transferred to absolute ethanol (Sinopharm) I for 5 min, absolute ethanol II for 5 min, and 75% ethanol for 5 min before being washed with water. The plant tissue sections were stained with safranin (Servicebio, Wuhan, China) for 1–2 h and subsequently washed with water. The sections were put into 50%, 70%, 80% gradient alcohol to decolorize. The sections were dyed in Fast Green (Servicebio) for 30–60 s, immersed in three-cylinder with absolute ethanol, and dehydrated. The slices were put in 1-Butanol (Sinopharm) and xylene for 5 min to be transparent, dried out slightly, and sealed with neutral gum (Sinopharm). Microscope inspection, image acquisition, and analysis were carried out.

### 4.9. RNA Extraction and Library Construction

Total RNA was extracted using the RNAiso Plus Kit (Takara Biomedical Technology (Beijing) Co., Ltd., Beijing, China). RNA integrity was evaluated with the Agilent RNA 6000 Pico Kit. Sequencing libraries were prepared using the NEBNext^®^ UltraTM RNA Library Prep Kit for Illumina^®^ (New England Biolabs, Ipswich, MA, USA), following the manufacturer’s instructions, and index codes were incorporated to assign sequences to individual samples. Sequencing was conducted on an Illumina NovaSeq platform, producing 150 bp paired-end reads.

### 4.10. Reads Processing and Gene Ontology Analysis

The data were analyzed using the free online Majorbio Cloud Platform (www.majorbio.com). Raw data processing involved modules that integrated the Fastx Toolkit, SeqPrep, and Sickle. Clean data were obtained by trimming adapter sequences, removing low-quality reads, and filtering short reads.

Transcript abundance was quantified as transcripts per million reads (TPM) using RSEM. Differential gene expression analysis between each pair of samples was conducted with a *p*-value threshold of <0.05, applying false discovery rate (FDR) correction via the Benjamini–Hochberg method. Significant differentially expressed genes (DEGs) were identified using the criterion |log₂FC| ≥ 1.

Gene Ontology (GO) and Kyoto Encyclopedia of Genes and Genomes (KEGG) pathway enrichment analyses of DEGs were performed using modules based on Goatool and KOBAS.

### 4.11. Data Analysis

All experimental data were analyzed using analysis of variance (ANOVA) in SPSS Statistics 22 (SPSS Inc., Chicago, IL, USA). When significant differences were detected, means were compared using Tukey’s HSD test at the 0.05 or 0.01 significance level.

## 5. Conclusions

**Figure 6:**
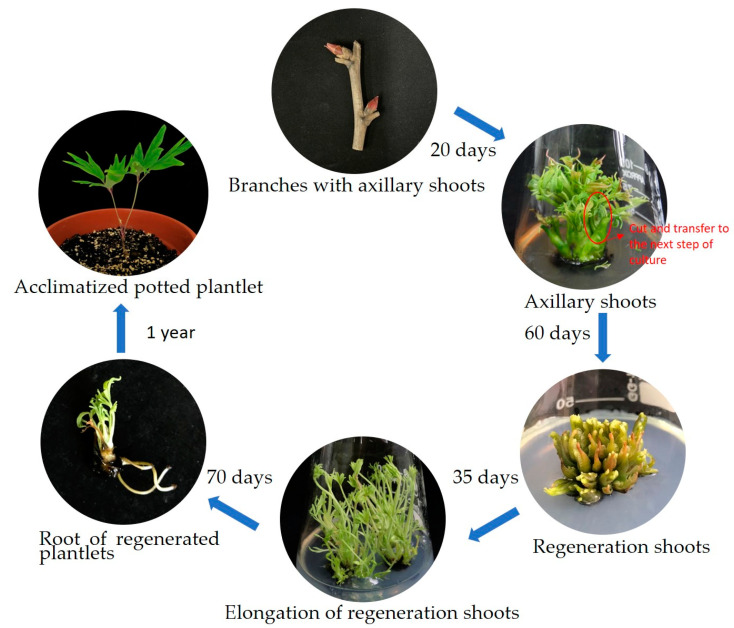
The whole flow scheme of a high-efficiency regeneration system using *P. ostii* ‘Feng Dan’ axillary shoots as explants.

An efficient in vitro regeneration system (Figure 6) was established using *P. ostii* ‘Feng Dan’ axillary shoots as explants to ensure the stable genetic inheritance of superior genotypes, achieving significant advancements in root induction. By optimizing the culture medium, it was discovered that Y3M medium with added BA and NAA greatly increased the number of adventitious shoots and their elongation quality. For root formation, the ideal medium was WPM with 3 mg/L IBA and 100 mg/L PG. Lignin content analysis, microscopic examination, and molecular signature results indicated that PG enhanced lignin biosynthesis, thus promoting in vitro rooting of *P. ostii*. This research not only offers an effective protocol for the micropropagation of *P. ostii*, but also highlights the essential role of PG in root induction, providing both theoretical and technical support for the large-scale production of tree peonies.

## Figures and Tables

**Table 1:** Effects of different combinations of PGRs on adventitious shoot induction of *P. ostii*.

Treatment	Plant Growth Regulators (mg/L)		Adventitious Shoot Induction Rate (%)	Average Adventitious Shoot Number
	BA	NAA		
1	0.5	0.05	41.67 ± 4.41 ^d^	3.88 ± 0.11 ^f^
2	0.5	0.1	56.67± 4.41 ^c^	4.18 ± 0.50 ^f^
3	0.5	0.2	31.67 ± 1.67 ^d^	2.10 ± 0.15 ^g^
4	1	0.05	76.67 ± 6.01 ^b^	9.88 ± 0.99 ^c^
5	1	0.1	96.67 ± 3.33 ^a^	16.03 ± 0.48 ^a^
6	1	0.2	88.33 ± 3.33 ^ab^	13.35 ± 0.46 ^b^
7	2	0.05	73.33 ± 4.41 ^b^	8.07 ± 0.27 ^de^
8	2	0.1	78.33 ± 7.26 ^b^	9.49 ± 0.75 ^cd^
9	2	0.2	86.67 ± 4.41 ^ab^	7.32± 0.26 ^e^

Means (±standard error) within a column followed by the same superscript letter were found not to be significantly different using Tukey’s multiple comparison test and *p* ≤ 0.05.

**Table 2:** Effect of different concentrations of BA and NAA on adventitious shoot elongation of *P. ostii*.

Treatment	Plant Growth Regulators (mg/L)		Adventitious Shoot Elongation Percentage (%)	Average Shoot Length (cm) (Length ≥ 0.5 cm)
	BA	NAA		
1	0.1	0.01	78.69 ± 1.05 ^b^	2.81 ± 0.04 ^c^
2	0.1	0.03	65.41 ± 1.18 ^de^	1.56 ± 0.06 ^f^
3	0.1	0.05	54.65 ± 1.25 ^g^	0.96 ± 0.03 ^h^
4	0.3	0.01	90.6 ± 1.08 ^a^	3.11 ± 0.05 ^b^
5	0.3	0.03	92.15 ± 1.06 ^a^	3.87 ± 0.06 ^a^
6	0.3	0.05	60.18 ± 1.43 ^ef^	2.29 ± 0.03 ^d^
7	0.5	0.01	55.82 ± 1.19 ^fg^	2.05 ± 0.05 ^e^
8	0.5	0.03	71.38 ± 1.15 ^c^	1.96 ± 0.04 ^e^
9	0.5	0.05	67.20 ± 1.29 ^cd^	1.30 ± 0.04 ^g^

Means (±standard error) within a column followed by the same superscript letter were found not to be significantly different using Tukey’s multiple comparison test and *p* ≤ 0.05.

**Table 3:** The macronutrient composition of the basal medium.

Basal Medium Ingredients	WPM (mg/L)	Y3M (mg/L)
NH_4_Cl	/	535
NH_4_NO_3_	400	/
KNO_3_	/	2020
CaCl_2_	72.5	222
Ca(NO_3_)_2_·4H_2_O	556	/
MgSO_4_·7H_2_O	370	247
KCl	/	1492
K_2_SO_4_	990	/
KH_2_PO_4_	170	354
KI	/	8.3
MnSO_4_·4H_2_O	22.5	11.2
ZnSO_4_·7H_2_O	8.6	7.2
H_3_BO_3_	6.2	3.1
CuSO_4_·5H_2_O	0.25	0.25
Na_2_MoO_4_·2H_2_O	0.25	0.24
CoCl_2_·6H_2_O	/	0.24
NiCl_2_·6H_2_O	/	0.024
FeSO_4_·7H_2_O	27.8	41.7
C_10_H_14_N_2_Na_2_O_8_	37.3	55.8
Nicotinic acid	0.5	0.5
Thiamine	1	0.05
Pyridoxine	0.5	0.05
Folic acid	/	0.05
Biotin	/	0.05
Glycine	2	1
Inositol	100	/

## Data Availability

Data are contained within the article and Supplementary Materials. The raw data of RNA-seq supporting the conclusions of the study have been deposited in China National GeneBank DataBase (CNGBdb, https://db.cngb.org/) and are accessible with the accession number CNP0001898.

## Supplementary Materials

The following supporting information can be downloaded at: https://www.mdpi.com/article/10.3390/plants13223200/s1, Figure S1: GO annotation of DEGs in stems (a) and leaves (b) with PG application; Figure S2: KEGG annotation of DEGs in stems (a) and leaves (b) with PG application; Figure S3: The functional protein association networks between phytohormones (Auxin, BR and CK) signal transduction and lignin biosynthesis pathways; Table S1: Categorization and abundance of the reads. Table S2: Number of annotated unigenes. Table S3: The expression strength of Unigens. Dataset S1: Details of GO annotation; Dataset S2: Details of KEGG annotation; Dataset S3: DEGs enriched in phytohormone signal transduction. The raw data of RNA-seq supporting the conclusions of the study have been deposited in China National GeneBank DataBase (CNGBdb, https://db.cngb.org/) and are accessible with the accession number CNP0001898.

## References

[1] Yang Y., Sun M., Li S., Chen Q., Teixeira da Silva J.A., Wang A., Yu X., Wang L.-S. (2020). Germplasm Resources and Genetic Breeding of Paeonia: A Systematic Review. Hortic. Res..

[2] Barreira J.C., Ferreira I.C., Oliveira M.B.P., Pereira J.A. (2008). Antioxidant Activities of the Extracts from Chestnut Flower, Leaf, Skins and Fruit. Food Chem..

[3] Wu K.-C., Lee D.-Y., Hsu J.-T., Cheng C., Lan J.-L., Chiu S.C., Cho D.Y., Hsu J.-L. (2021). Evaluations and Mechanistic Interrogation of Natural Products Isolated from *Paeonia suffruticosa* for the Treatment of Inflammatory Bowel Disease. Front. Pharmacol..

[4] Deng R., Gao J., Yi J., Liu P. (2022). Could peony seeds oil become a high-quality edible vegetable oil? The nutritional and phytochemistry profiles, extraction, health benefits, safety and value-added-products. Food Res. Int..

[5] Deng R., Gao J., Yi J., Liu P. (2022). Peony seeds oil by-products: Chemistry and bioactivity. Ind. Crops Prod..

[6] Jiang X., Yang C., Beta T., Liu S., Yang R. (2019). Phenolic Profile and Antioxidant Activity of the Edible Tree Peony Flower and Underlying Mechanisms of Preventive Effect on H_2_O_2_-Induced Oxidative Damage in Caco-2 Cells. Foods.

[7] Zhang H., Li X., Ke W., Wang M., Liu P., Wang X., Deng R. (2016). Antioxidant Activities and Chemical Constituents of Flavonoids from the Flower of *Paeonia ostii*. Molecules.

[8] Ekiert H., Klimek-Szczykutowicz M., Szopa A. (2022). *Paeonia* × *suffruticosa* (Moutan Peony)—A Review of the Chemical Composition, Traditional and Professional Use in Medicine, Position in Cosmetics Industries, and Biotechnological Studies. Plants.

[9] Zhang X., Zhai Y., Yuan J., Hu Y. (2019). New insights into Paeoniaceae used as medicinal plants in China. Sci. Rep..

[10] Peng X.-N., Zhou Y., Liu Y.-X., Huo Y.-L., Ren J.-Y., Bai Z.-Z., Zhang Y.-L., Tang J.-J. (2023). Neuroprotective potential of phytochemicals isolated from *Paeonia ostii* ‘Feng Dan’ stamen. Ind. Crops Prod..

[11] Wang F. (2019). Common tree peony propagation methods and cultivation techniques. Mod. Agric. Sci. Technol..

[12] Cheng F. (2007). Advances in the breeding of tree peonies and a cultivar system for the cultivar group. Int. J. Plant Breeding.

[13] Zeng D., Yin W., Zhao X., Wang H. (2000). Propagation of Chinese tree peony (*Paeonia suffruticosa* Andr). J. Beijing For. Univ..

[14] Yuan J., Jiang S., Jian J., Liu M., Yue Z., Xu J., Li J., Xu C., Lin L., Jing Y. (2022). Genomic basis of the giga-chromosomes and giga-genome of tree peony *Paeonia ostii*. Nat. Commun..

[15] Wen S.S., Chen L., Tian R.N. (2020). Micropropagation of tree peony (*Paeonia* sect. *Moutan*): A review.. Plant Cell Tissue Organ Cult. (PCTOC).

[16] Wen S.-S., Cheng F.-Y., Zhong Y., Wang X., Li L.-Z., Zhang Y.-X., Qiu J.-M. (2016). Efficient protocols for the micropropagation of tree peony (*Paeonia suffruticosa* ‘Jin Pao Hong’, *P. suffruticosa* ‘Wu Long Peng Sheng’, and *P.× lemoinei* ‘High Noon’) and application of arbuscular mycorrhizal fungi to improve plantlet establishment. Sci. Hortic..

[17] Liu R., Xue Y., Ci H., Gao J., Wang S., Zhang X. (2022). Establishment of highly efficient plant regeneration of *Paeonia ostii* ‘Fengdan’ through optimization of callus, adventitious shoot, and rooting induction. Hortic. Plant J..

[18] Du Y., Cheng F., Zhong Y. (2020). Induction of direct somatic embryogenesis and shoot organogenesis and histological study in tree peony (*Paeonia* sect. *Moutan*). Plant Cell Tissue Organ Cult. (PCTOC).

[19] Teixeira da Silva J.A., Shen M., Yu X. (2012). Tissue culture and micropropagation of tree peony (*Paeonia suffruticosa* Andr.). J. Crop Sci. Biotechnol..

[20] Zhu X., Li X., Ding W., Jin S., Wang Y. (2018). Callus induction and plant regeneration from leaves of peony. Hortic. Environ. Biotechnol..

[21] Chen X., Ye C., Yang H., Ji W., Xu Z., Ye S., Wang H., Jin S., Yu C., Zhu X. (2022). Callogenesis and Plant Regeneration in Peony (*Paeonia* × *suffruticosa*) Using Flower Petal Explants. Horticulturae.

[22] Wang H., van Staden J. (2001). Establishment of in vitro cultures of tree peonies. S. Afr. J. Bot..

[23] Wang X., Cheng F., Zhong Y., Wen S., Li L., Huang N. (2016). Establishment of in vitro Rapid Propagation System for Tree Peony (*Paeonia ostii*). Sci. Silvae Sincae.

[24] Gómez-Kosky R., Armas P.M., Calimano M.B., Villegas A.B., Otero Y., Jaramillo D.N., Ferreiro J.Á., Daniels D.D., Pérez L.P. (2021). Effect of Phloroglucinol on in Vitro Rooting of Sugarcane (*Saccharum* spp. cv C90-469). Sugar Tech.

[25] Tchouga A.O., Deblauwe V., Mouafi Djabou S.A., Forgione G., Hanna R., Niemenak N. (2020). Micropropagation and effect of phloroglucinol on rooting of *Diospyros crassiflora* Hiern. HortScience.

[26] Petti C. (2020). Phloroglucinol mediated plant regeneration of ornithogalum dubium as the sole “hormone-like supplement” in plant tissue culture long-term experiments. Plants.

[27] Teixeira da Silva J.A., Dobránszki J., Ross S. (2013). Phloroglucinol in plant tissue culture. In Vitro Cell. Dev. Biol. Plant.

[28] Yan X., Wang K., Zheng K., Zhang L., Ye Y., Qi L., Zhu M. (2023). Efficient organogenesis and taxifolin production system from mature zygotic embryos and needles in larch. For. Res..

[29] Suman Kumar J.N.J. (2015). Phloroglucinol Plays Role in Shoot Bud Induction and In Vitro Tuberization in Tinospora Cordifolia—A Medicinal Plant with Multi-Therapeutic Application. Adv. Tech. Biol. Med..

[30] Londe L., Vendrame W., Oliveira A., Sanaey M., Costa A. (2017). Phloroglucinol is Effective for in vitro Growth and Multiplication of *Musa accuminata* Cv. Grand Naine Shoots and Roots. J. Adv. Biol. Biotechnol..

[31] Zhu F., Wang S., Xue J., Li D., Ren X., Xue Y., Zhang X. (2018). Morphological and physiological changes, and the functional analysis of PdSPL9 in the juvenile-to-adult phase transition of *Paeonia delavayi*. Plant Cell Tissue Organ Cult. (PCTOC).

[32] Xu L., Cheng F., Zhong Y. (2022). In Vitro Immature Embryo Culture of *Paeonia ostii* ‘Feng Dan’. HortScience.

[33] Xu L., Cheng F., Zhong Y. (2022). Efficient Plant Regeneration via Meristematic Nodule Culture in *Paeonia ostii* ‘Feng Dan’. Plant Cell Tissue Organ Cult. (Pctoc).

[34] Sardans J.A.-O., Peñuelas J.A.-O. (2021). Potassium Control of Plant Functions: Ecological and Agricultural Implications. Plants.

[35] Asif M., Yılmaz Ö., Öztürk L. (2017). Potassium Deficiency Impedes Elevated Carbon Dioxide-induced Biomass Enhancement in Well Watered or Drought-stressed Bread Wheat. J. Plant Nutr. Soil Sci..

[36] Saleem M.F., Aown M., Raza S., Ahmad S., Shahid A.M. (2016). Understanding and Mitigating the Impacts of Drought Stress in Cotton—A Review. Pak. J. Agric. Sci..

[37] Zhou H., Sun J., Zheng K., Zhang X., Yao Y., Zhu M. (2024). Efficient Plantlet Regeneration from Branches in *Mangifera indica* L.. Plants.

[38] Teixeira da Silva J.A., Gulyás A., Magyar-Tábori K., Wang M.R., Wang Q.C., Dobránszki J. (2019). In vitro tissue culture of apple and other Malus species: Recent advances and applications. Planta.

[39] Pérez L.P., Montesinos Y.P., Olmedo J.G., Rodriguez R.B., Sánchez R.R., Montenegro O.N., Escriba R.C.R., Daniels D., Gómez-Kosky R. (2016). Effect of phloroglucinol on rooting and in vitro acclimatization of papaya (*Carica papaya* L. var. *Maradol Roja*). In Vitro Cell. Dev. Biol. Plant.

[40] Lerin J., Ribeiro Y.R.S., de Oliveira T.R., Silveira V., Santa-Catarina C. (2021). Histomorphology and proteomics during rooting of in vitro shoots in *Cariniana legalis* (Lecythidaceae), a difficult-to-root endangered species from the Brazilian Atlantic Forest. Plant Cell Tissue Organ Cult..

[41] Murphy R., Adelberg J. (2021). Physical factors increased quantity and quality of micropropagated shoots of *Cannabis sativa* L. in a repeated harvest system with ex vitro rooting. In Vitro Cell. Dev. Biol. Plant.

[42] Naidoo D., Aremu A.O., Van Staden J., Finnie J.F. (2017). In vitro plant regeneration and alleviation of physiological disorders in Scadoxus puniceus. S. Afr. J. Bot..

[43] Nair R.B., Bastress K.L., Ruegger M.O., Denault J.W., Chapple C.J.T.P.C. (2004). The *Arabidopsis thaliana REDUCED EPIDERMAL FLUORESCENCE1* gene encodes an aldehyde dehydrogenase involved in ferulic acid and sinapic acid biosynthesis. Plant Cell.

[44] Lloyd G., McCown B. (1980). Commercially-feasible micropropagation of mountain laurel, *Kalmia latifolia*, by use of shoot-tip culture. Comb. Proc. Int. Plant Propagators’ Soc..

[45] Eeuwens C. (1976). Mineral requirements for growth and callus initiation of tissue explants excised from mature coconut palms (*Cocos nucifera*) and cultured in vitro. Physiol. Plant..

