# Supplementary material for: An Efficient In Vitro Regeneration Protocol and the Feature of Root Induction with Phloroglucinol in Paeonia ostii
Source: Plants (Basel). 2024 Nov 14;13(22):3200. doi: 10.3390/plants13223200 (PMC11598103; doi:10.3390/plants13223200)
Supplement: Supplementary file 1 [file plants-13-03200-s001.zip › plants-3282193-Supplementary File/Supplementary Tables.docx]

| **Table S1 Categorization and abundance of the reads** | | | | | | | | | |
| --- | --- | --- | --- | --- | --- | --- | --- | --- | --- |
| Sample | | Raw reads | Clean reads^1^ | Error rate(%)^2^ | Q20(%) | Q30(%) | GC content(%)^3^ | Mapped reads | |
|  |  |  |  |  |  |  |  | Total mapped | Mapped ratio |
| Leaf | C1 | 132,544,634 | 132,495,166 | 0.0249 | 98.11 | 94.16 | 45.79 | 95,661,716 | 72.20% |
|  | C2 | 77,030,912 | 76,886,516 | 0.0282 | 96.76 | 91.28 | 45.22 | 58,152,514 | 75.63% |
|  | C3 | 110,098,282 | 110,041,662 | 0.0239 | 98.52 | 95.25 | 45.42 | 82,478,592 | 74.95% |
|  | T1 | 116,806,140 | 116,706,310 | 0.0246 | 98.22 | 94.46 | 45.77 | 84,943,586 | 72.78% |
|  | T2 | 106,648,528 | 106,605,200 | 0.0246 | 98.24 | 94.50 | 45.23 | 81,191,788 | 76.16% |
|  | T3 | 113,023,146 | 112,968,382 | 0.0242 | 98.41 | 94.93 | 45.71 | 83,047,830 | 73.51% |
| Stem | C1 | 104,276,630 | 104,225,954 | 0.0241 | 98.43 | 95.02 | 45.53 | 79,412,268 | 76.19% |
|  | C2 | 128,852,986 | 128,806,922 | 0.0242 | 98.37 | 94.88 | 45.72 | 98,940,144 | 76.81% |
|  | C3 | 105,920,320 | 105,867,284 | 0.0246 | 98.23 | 94.51 | 45.96 | 78,128,404 | 73.80% |
|  | T1 | 119,461,894 | 119,413,648 | 0.0245 | 98.26 | 94.60 | 45.92 | 88,721,758 | 74.30% |
|  | T2 | 122,519,014 | 122,484,238 | 0.0244 | 98.31 | 94.70 | 45.73 | 91,779,324 | 74.93% |
|  | T3 | 125,312,318 | 125,261,680 | 0.0244 | 98.27 | 94.66 | 45.90 | 93,808,032 | 74.89% |

1. Reads that remained after filtering out low quality reads from raw data.

2. Error rate based on clean data.

3. GC content based on clean data.

| **Table S2 Number of annotated unigenes** | | |
| --- | --- | --- |
| **Samples** | | **No of Unigenes** |
| Leaf | C1 | 42,320 |
|  | C2 | 41,553 |
|  | C3 | 42,396 |
|  | T1 | 43,213 |
|  | T2 | 38,709 |
|  | T3 | 39,797 |
| Stem | C1 | 38,321 |
|  | C2 | 39,298 |
|  | C3 | 37,852 |
|  | T1 | 39,281 |
|  | T2 | 37,173 |
|  | T3 | 39,512 |

| **Table S3 The expression strength of Unigens** | | | | | |
| --- | --- | --- | --- | --- | --- |
| Sample | | Expression strength | | | |
|  |  | (0, 1] | (1, 10] | (10, 100] | (100, +∞) |
| Leaf | C1 | 73.10 | 19.79 | 6.32 | 0.78 |
|  | C2 | 72.46 | 19.81 | 6.84 | 0.89 |
|  | C3 | 73.22 | 19.31 | 6.60 | 0.87 |
|  | T1 | 70.05 | 22.54 | 6.67 | 0.74 |
|  | T2 | 70.19 | 20.04 | 8.60 | 1.17 |
|  | T3 | 73.47 | 18.83 | 6.81 | 0.89 |
| Stem | C1 | 70.27 | 20.06 | 8.45 | 1.22 |
|  | C2 | 72.42 | 18.99 | 7.50 | 1.08 |
|  | C3 | 71.99 | 19.51 | 7.47 | 1.03 |
|  | T1 | 74.35 | 17.84 | 6.78 | 1.04 |
|  | T2 | 73.24 | 18.26 | 7.33 | 1.17 |
|  | T3 | 74.85 | 17.53 | 6.58 | 1.04 |
