# Supplementary figures and images for: An Efficient In Vitro Regeneration Protocol and the Feature of Root Induction with Phloroglucinol in Paeonia ostii
Source: Plants (Basel). 2024 Nov 14;13(22):3200. doi: 10.3390/plants13223200 (PMC11598103; doi:10.3390/plants13223200)

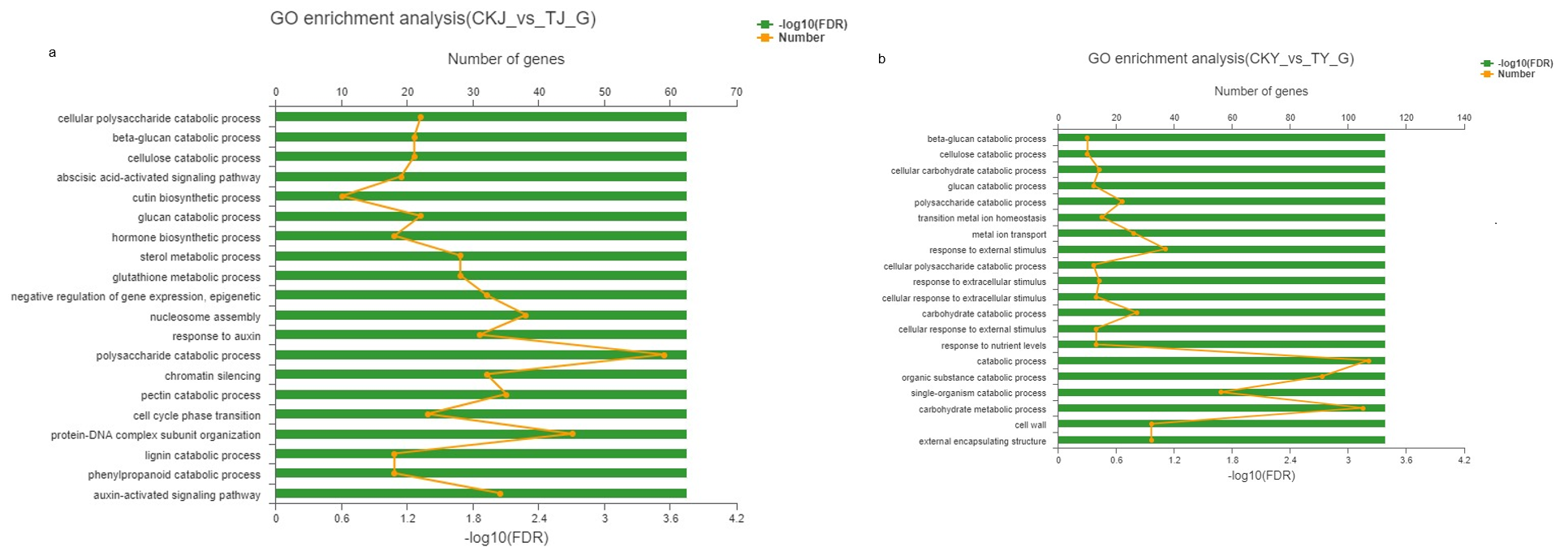

Supplement: Supplementary file 1 [file plants-13-03200-s001.zip › plants-3282193-Supplementary File/Figure S1.tif]

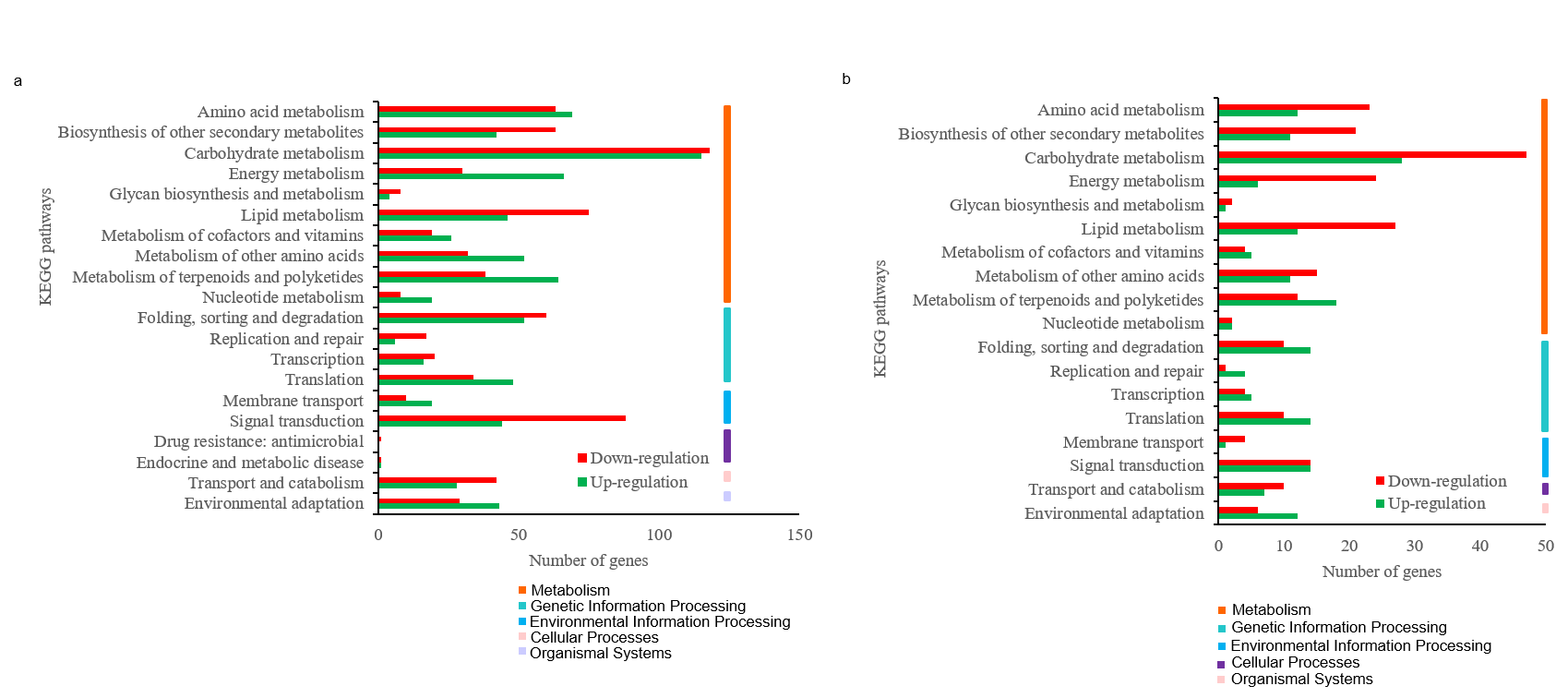

Supplement: Supplementary file 1 [file plants-13-03200-s001.zip › plants-3282193-Supplementary File/Figure S2.tif]

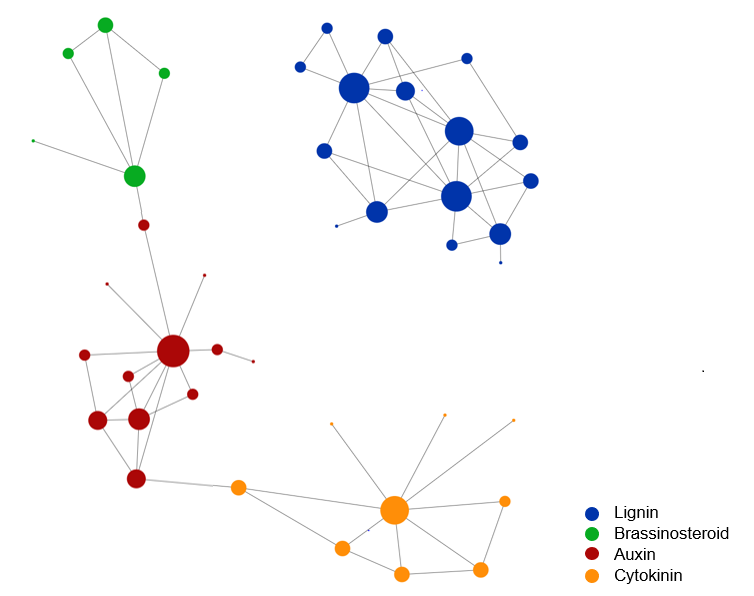

Supplement: Supplementary file 1 [file plants-13-03200-s001.zip › plants-3282193-Supplementary File/Figure S3.tif]
